# Pulmonary Embolism in a Sarcoidosis Patient Double Heterozygous for Methylenetetrahydrofolate Reductase Gene Polymorphisms and Factor V Leiden and Homozygous for the D-Allele of Angiotensin Converting Enzyme Gene

**DOI:** 10.1155/2015/606805

**Published:** 2015-08-11

**Authors:** Nadim El-Majzoub, Rami Mahfouz, Nadim Kanj

**Affiliations:** ^1^Department of Pathology and Laboratory Medicine, American University of Beirut Medical Center, P.O. Box 11-0236, Riad El Solh, Beirut 110 72020, Lebanon; ^2^Division of Pulmonary and Critical Care Medicine, Department of Internal Medicine, American University of Beirut Medical Center, P.O. Box 11-0236, Riad El Solh, Beirut 110 72020, Lebanon

## Abstract

Sarcoidosis is a multisystem granulomatous disease of unknown etiology and pathogenesis. It presents in patients younger than 40 years of age. The lungs are the most commonly affected organ. Till the present day, there is no single specific test that will accurately diagnose sarcoidosis; as a result, the diagnosis of sarcoidosis relies on a combination of clinical, radiologic, and histologic findings. Patients with sarcoidosis have been found to have an increased risk of pulmonary embolism compared to the normal population. MTHFR and factor V Leiden mutations have been reported to increase the risk of thrombosis in patients. We hereby present a case of a middle aged man with sarcoidosis who developed a right main pulmonary embolism and was found to be double heterozygous for methylenetetrahydrofolate reductase gene polymorphisms and factor V Leiden and homozygous for the D-allele of the angiotensin converting enzyme gene.

## 1. Introduction

Sarcoidosis is a multisystem granulomatous disease of yet unknown etiology and pathogenesis. It usually presents in patients younger than 40 years of age with a peak incidence between 20 and 29 years. The disease may be asymptomatic or chronic. Sarcoidosis presents with nonspecific symptoms. The lungs are the most commonly affected organ with other organs being affected but to a lower extent. Patients with pulmonary sarcoidosis usually present with cough, shortness of breath, and chest pain. Staging of pulmonary sarcoidosis is based on chest X-ray imaging with 5 stages described [1, 2]. Till the present day, there is no single specific test that will accurately diagnose sarcoidosis; as a result, the diagnosis of sarcoidosis relies on a combination of clinical, radiologic, and histologic findings. Patients with sarcoidosis have been found to have a twofold increased risk of developing pulmonary embolism (PE) compared to the normal population [3]. Methylenetetrahydrofolate reductase (MTHFR) gene polymorphisms and factor V Leiden have been documented to increase a person's risk of developing venous thromboembolism (VTE). MTHFR gene polymorphisms and factor V Leiden are common in the Lebanese community with a prevalence of the heterozygous state of 39.73% and 14%, respectively [4–6]. We hereby present a case of a middle aged man with sarcoidosis who developed a right main pulmonary artery embolism and was found to be double heterozygous for MTHFR gene polymorphisms and factor V Leiden and homozygous for the D-allele of the angiotensin converting enzyme gene.

## 2. Case History

A 60-year-old governmental employee male with stage IV sarcoidosis on corticosteroids for many years presented to our emergency department (ED) due to a 2-month history of episodic low grade fever and dyspnea. The patient is a nonsmoker and was diagnosed with sarcoidosis thirteen years ago after excluding other diagnoses based on chest X-ray, a negative Mantoux skin test, pulmonary function test showing severe restrictive disease, and a lung biopsy showing pulmonary fibrosis secondary to sarcoidosis (April 2000) as a result of which he was started on high dose steroids. In October 2003, bronchoscopy revealed narrowing of the right upper lobe and right lower lobe segment which was balloon dilated to 9 mm. In September 2005 and as part of a workup for infertility, a spermogram showed oligospermia. Ultrasound Doppler for testicles showed bilateral scrotal varices and the patient underwent a bilateral varicocelectomy.

In the ED, physical exam was unremarkable except for decreased air entry over the right upper lobe. Initial laboratory investigations including a complete blood count and electrolytes were all within normal range (WBC: 9,100/cc·mm [4,000–11,000], hemoglobin 14.3 g/dL [13–18], mean corpuscular volume 83 fL [80–94], platelets 213,000/cu·mm [150,000–400,000] with a white blood cell differential showing polymorphonuclear cells 85% [40–65], lymphocytes 9% [25–40], monocytes 5% [2–8], eosinophils 1% [0–4], creatinine 0.8 [0.5–1.0], sodium 140 mmoL/L [135–145], potassium 4 mmoL/L [3.5–5.1], chloride 101 [98–109], carbon dioxide 29 [24–30], magnesium 2.3 [1.6–2.5], calcium 9.6 [8.5–10.5], and inorganic phosphate 2.6 [2.7–4.8]). Prothrombin time was 12.7 seconds, INR 1.10 [0.85–1.20], activated prothrombin time 29 seconds, and Troponin T 0.009 [0–0.030].

**Figure 1:**
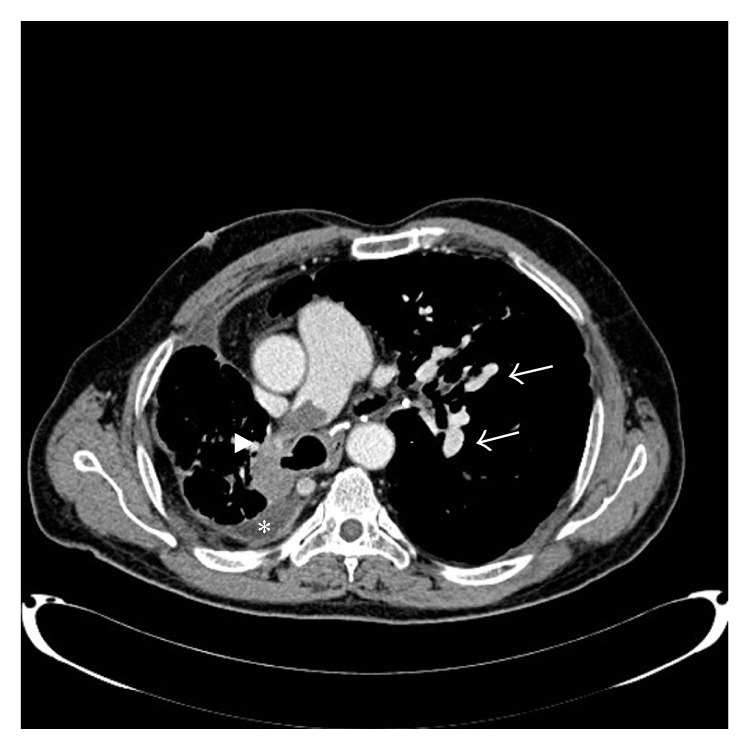
Computed tomography scan of the chest with contrast showing a pulmonary embolus in the right main pulmonary artery (arrow head), with calcified lymph nodes (arrow), in addition to right pleural effusion (asterisk).

Computed tomography (CT) of the chest with contrast showed a pulmonary embolus (PE) in the right main pulmonary artery, with stable fibrotic lung changes and calcified lymph nodes, in addition to a mild right pleural effusion (Figure 1). CT of abdomen/pelvis was unremarkable. A diagnostic pleural tap (100 mL of fluid recovered) showed a neutrophil predominant exudate (red blood cells: 18,400/cu·mm, white blood cells: 280/cu·mm with 98% polymorphonuclear cells and 2% monocytes) and culture was negative for microorganisms. Semiquantitative nested real-time polymerase chain reaction on the GeneExpert platform (Cepheid) showed absence of mycobacterial tuberculosis complex DNA in the pleural fluid. Extremity venous duplex scan was negative for deep venous thrombosis. As a result, the patient was started on low molecular weight heparin and warfarin. A transthoracic echocardiogram showed an ejection fraction of 45–49% with right ventricular apical akinesia. Electrocardiogram revealed a right bundle branch block.

Genetic testing using the Cardiovascular Disease (CVD) Panel StripAssay (ViennaLab, Austria) revealed the patient to be heterozygous for beta-fibrinogen −455 G>A, factor V Leiden (G1691A), and MTHFR gene polymorphisms (both C677T and A1298C) and homozygous for angiotensin converting enzyme (ACE) D-allele. He had a normal factor II, factor XIII, ApoB, ApoE, plasminogen activator inhibitor-1, and glycoprotein IIIa L33P. Antithrombin III was 73% [83–128]. Lupus anticoagulant was negative. Protein C was 46% [69–134]. Protein S was 69.2% [65–140] and had a mildly elevated homocysteine level 15.6 *μ*moL/L [5–15]. He underwent a cardiac magnet resonance imaging (Ingenia 3T Philips MRI scanner) that showed features of pulmonary hypertension with evidence of restrictive cardiomyopathy due to sarcoidosis. The patient was advised to do a 24-hour Holter monitoring in 1 year to follow up on his right bundle branch block (RBBB).

## 3. Discussion

Sarcoidosis is also known as Besnier-Boeck disease. Professor Cæsar Peter Møller Boeck, a Norwegian dermatologist, was the first to describe the skin changes associated with sarcoidosis in 1899 and later described involvement of other organs of the body with the disease. Boeck later published his findings about sarcoidosis in an article titled “Multiple benign sarcoid of the skin,” named so for their resemblance to malignant sarcoma.

Sarcoidosis is a systemic disease capable of affecting many organs at the same time, with the lungs being the most commonly affected. The cause and pathogenesis of this disease are still unknown. In the United States, the prevalence of sarcoidosis has been steadily increasing between 1995 (164 per 100,000) and 2010 (330 per 100,000) [7]. Unfortunately, the prevalence of this disease in Lebanon is still lacking. Based on a study from Dhahran Health Center, Saudi Arabia, the estimated prevalence of sarcoidosis in the Eastern region of Saudi Arabia is 13 per 100,000 [8]. Studies about the predilection of sarcoidosis to females have been controversial. According to a study in the Eastern region of Saudi Arabia the male to female ratio was comparable [8], while, according to a Turkish study, women are affected more often than men and diagnosed at an older mean age [9].

Radiologic staging of sarcoidosis was first described five decades ago by Scadding [1] and is still used with the addition of stage 0 for normal chest X-ray [2] (Table 1 describes the five different stages of sarcoidosis).

Crawshaw and colleagues [3] conducted a retrospective cohort analysis comparing patients with sarcoidosis and a matched group of patients without sarcoidosis and proved that the former group had a twofold increased risk of PE compared to the latter group. These findings were confirmed by Swigris and colleagues [10] who showed that, compared to the general population, sarcoidosis patients have an increased risk of PE irrespective of age, gender, and race. On the other hand, among patients with sarcoidosis, there was no difference in the risk of PE based on gender or race. Sarcoidosis patients with PE were more likely to have pulmonary hypertension compared to those with sarcoidosis but without PE but less likely to have cardiac diseases including myocardial ischemia, myocardial infarction, congestive heart failure, and cardiomyopathy.

MTHFR and factor V Leiden mutations have been reported to increase the risk of thrombosis. The prevalence of heterozygous MTHFR gene polymorphisms in the normal Lebanese population is 39.73% [6]. According to a recent meta-analysis on 11,000 cases and 21,000 controls [11], patients with homozygous C677T MTFHR were not at increased risk of VTE. After stratifying patients with homozygous MTHFR gene polymorphisms based on gender, men were found to have increased risk of VTE. On the other hand, the prevalence of factor V Leiden in the normal Lebanese population is 12.38% [4], and according to the aforementioned meta-analysis, heterozygous factor V Leiden patients are at increased risk of VTE [11]. Even though it would be expected that being double heterozygous for MTHFR gene polymorphisms and factor V Leiden would put our patient at an increased risk of VTE, the meta-analysis failed to show an interaction between the C677T MTHFR variant and factor V Leiden for VTE development.

Rebeiz and colleagues [12] have described the first case in the literature of a young Lebanese man being diagnosed with sarcoidosis after presenting with pulmonary embolism, diffuse arterial thrombosis of the lower extremities, and a left renal infarct. This patient was found to be homozygous for both MTHFR and factor XIII and heterozygous for factor II gene polymorphisms along with hyperhomocysteinemia. Ungprasert and Srivali [13] reported an unusual case of sarcoidosis presenting with superior mesenteric vein thrombosis with a negative thrombophilia profile upon investigation.

The ACE gene is located on the long arm of chromosome 17 at position 23.3. Even though ACE gene polymorphisms have been proven to be associated with an increased risk for cardiovascular disease [14], there is no evidence that it is associated with an increased risk of sarcoidosis [15]. Interestingly, the same study showed that there is a higher frequency of the D-allele in sarcoidosis patients compared to healthy subjects.

Some of the postulated causes of increased thrombosis risk in sarcoidosis patients are local venous compression, local tissue hypercoagulable state, and granulomatous thrombophlebitis. With the growing number of patients with sarcoidosis incidentally found to harbor an abnormal thrombophilia profile, it may become necessary in the near future to screen sarcoidosis patients before developing any hypercoagulable condition and thus decrease future morbidity.

In summary, we have described a case of sarcoidosis complicated by right main pulmonary artery embolism. Coexistence of sarcoidosis and double heterozygous MTHFR gene polymorphisms and factor V Leiden is rare. Having seen that being heterozygous for MTHFR gene polymorphisms is not a significant factor in developing PE, we propose that factor V Leiden was the culprit behind our patient's thrombotic presentation.

## Figures and Tables

**Table 1:** Staging of pulmonary sarcoidosis based on chest X-ray.

Staging	Description
0	Absence of chest X-ray abnormalities

1	Bilateral hilar lymphadenopathy that may be accompanied by right paratracheal and aortopulmonary window adenopathy

2	Bilateral hilar lymphadenopathy and parenchymal infiltration with a bilateral symmetric micronodular or reticulonodular pattern with predominant perihilar distribution, in middle and upper lung fields

3	Parenchymal infiltration without hilar adenopathy

4	Fibrosis with evidence of reticular pattern with traction bronchiectasis, masses causing architectural distortions, or honeycomb cysts, predominantly in the upper fields
